# Phenylpropanoids as master regulators: state of the art and perspectives in common bean (*Phaseolus vulgaris*)

**DOI:** 10.3389/fpls.2014.00336

**Published:** 2014-07-17

**Authors:** Oswaldo Valdés-López, Georgina Hernández

**Affiliations:** ^1^Unidad de Morfología y Función, Laboratorio de Bioquímica, Facultad de Estudios Superiores Iztacala, Universidad Nacional Autónoma de MéxicoTlalnepantla, Mexico; ^2^Genómica Funcional de Eucariotes, Centro de Ciencias Genómicas, Universidad Nacional Autónoma de MéxicoCuernavaca, México

**Keywords:** common bean, *Phaseolus vulgaris*, phenypropanoids, genetic mapping, transcription factors

Like other organisms, plants have to deal with a dynamic environment. To respond and adapt to the environmental conditions plants rely in a diverse battery of cell-surface protein-receptors and secondary metabolites. Phenylpropanoids are metabolites required for this big task, they regulate a wide range of physiological process, such as pigmentation of flowers and fruits, seed dispersal through attracting pollinators, auxin transport and UV-B protection (Peer and Murphy, 2007; Tanaka et al., 2008; Agati and Tattini, 2010; Vogt, 2010). Likewise, these secondary metabolites are critical to establish symbiotic interactions as well as to fight against pathogens (Dixon et al., 2002; Cooper, 2004). Besides these roles *in planta*, phenylpropanoids play an important role in the human health. For example, different flavonoids have neuroprotective, anti-inflammatory, analgesic, bacterial and anti-fungicidal activity (Figure 1; Yu and Jez, 2008).

**Figure 1:**
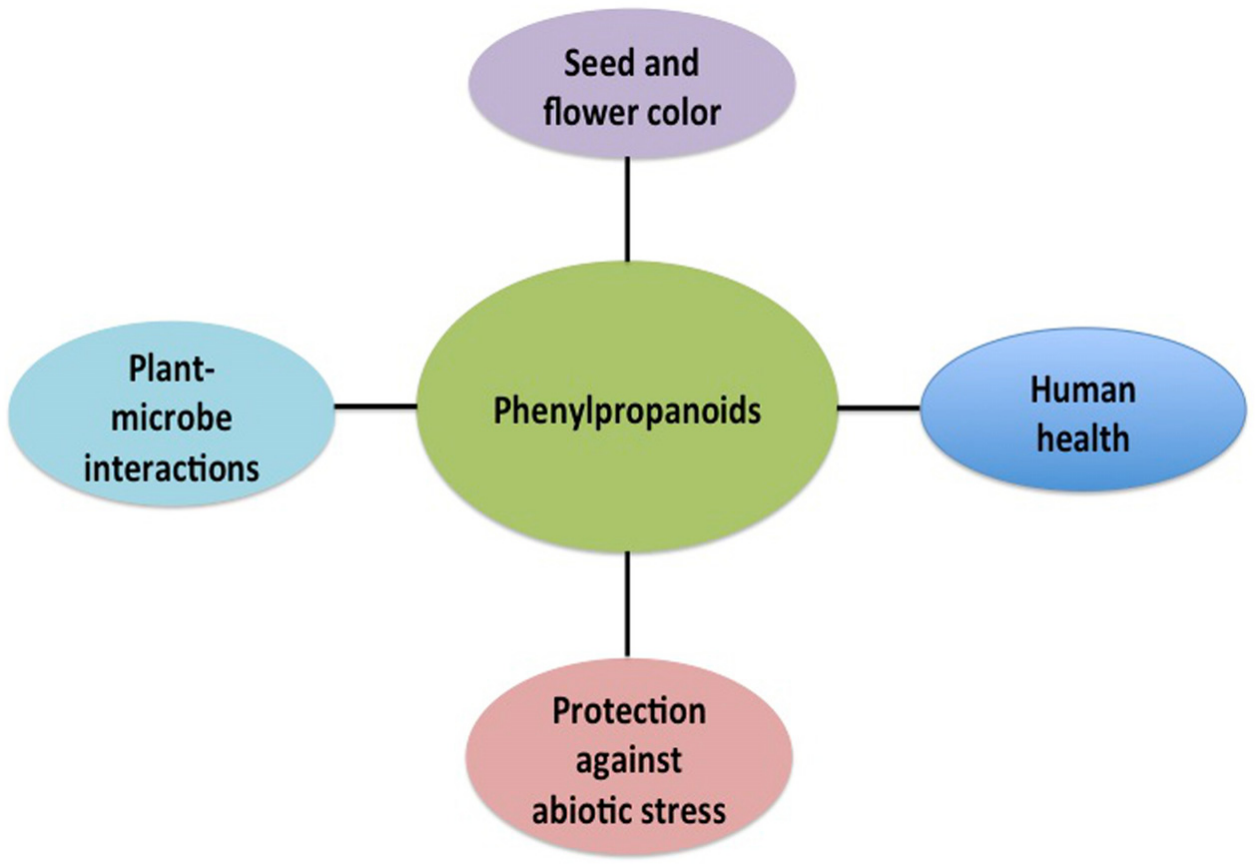
**Phenylpropanoid derivatives control different physiological aspects at *planta* level and play an important role in the human health**.

One of the most exiting examples about the relevance of phenylpropanoid *in planta* and human health levels is the common bean (*Phaseolus vulgaris*). In one hand, this legume uses different flavonoids to establish a symbiotic interaction with the N_2_-fixing soil bacteria collectively known as rhizobia. Through this interaction, common bean obtains nitrogen for its own metabolic requirements and this in turn is delivered to other organisms, including humans. In the other hand, common bean grains, besides being the main source of protein and fiber for human consumption, provide a variety of flavonoids, isoflavonoids and lignans with potential medical properties (Broughton et al., 2003).

Most of our knowledge about the phenylpropanoid synthesis and the genetic control of this pathway come from biochemical and genetic studies on the model plant *Arabidopsis thaliana* (Vogt, 2010). Despite the ecological and dietary relevance of common bean, our knowledge about the genetic control of the phenylpropanoids in this legume is scarce. To fill-up this gap, Reinprecht et al. (2013) used comparative genomics analyses and traditional PCR-based cloning to identify 46 structural and regulatory genes from the common bean phenylpropanoid pathway. The identified genes would play roles in: general phenylpropanoid pathway (*PAL*, *C4H*, and *4CL*), lignin/lignan- (*CCR*, *CAD*, *C3H*), flavonoid/anthocyanin- (*CHS*, *CHI*, *CHR*, *F3H*) or isoflavonoid (*IFS*, *IFR*, *7IOMT*) biosynthesis. Interestingly, with these experimental approaches, Reinprecht and colleagues were able to identify the transcription factors *LIM*, *KAP-2*, *HD*, *WIP*, and *Myb15*, all of them with a potential role in the transcriptional control of the phenylpropanoid pathway. These results provide a significant insight in our knowledge about the genetic control of the phenylpropanoid pathway in common bean. Furthermore, this study provides two additional aspects: (1) a large repertory of candidate genes for reverse genetic analyses that can lead us to understand their role in the biosynthesis of these secondary metabolites, and (2) a variety of marker genes that can be used for plant breeding programs in common bean.

Mapping genes into the genome is a required step for plant breeding programs. For this, it is also important to know the genome sequence of the plant of interest. For several years, the lack of the common bean genome sequence was one of the main limitations to do a deep genetic analysis. Recently, the common bean genome sequence was reported (Schmutz et al., 2014). This together with the known soybean genome sequence (Schmutz et al., 2010) opens the gates to perform detailed comparative analysis. Reinprecht et al. (2013) harnessed the close genetic relatedness between common bean and soybean to performed a comparative mapping *in silico* and were able to map the 46 identified-phenylpropanoid genes into some of the 11 chromosomes of common bean. For example, they mapped *CHS*, *Myb4*, *PAL3*, and *LIM* in the common bean chromosome *Pv*2, *Pv*6, *Pv*8, *Pv*9, respectively. Perhaps one of the most interesting results of this mapping analysis is the fact that most of the identified transcription factor genes were mapped in the chromosome *Pv*10. This finding is relevant considering that there are several cases where most of the genes that control a particular pathway are located in one chromosome. For example, several genes that control the symbiotic interaction between *Medicago truncatula* and rhizobia are located in the chromosome 5 (Ané et al., 2002; Arrighi et al., 2006; Horváth et al., 2011).

Knowing the structural and regulatory genes of the phenylpropanoids pathway will certainly help to understand how these secondary metabolites regulate different physiological processes. Likewise, having their genomic location will contribute to develop breeding programs in common bean. However, it is important to realize that several aspects have to be improved before carrying this out. Perhaps the two most important aspects are: (1) increase the number of marker genes and saturate the common bean genome; (2) develop powerful common bean mapping populations, for instance Nested Association Mapping lines. Indeed, it is widely documented that this kind of mapping population provides a high resolution in mapping analysis (Poland et al., 2011). If we consider that: (1) common bean has a vast genetic diversity (even greater than soybean diversity) and (2) Next Generation Sequencing technology has decreased the cost to get the whole genome sequence of any organism; these two aspect are feasible with no problem. Having a high resolution genetic map and a powerful mapping population will help us to accurately map these genes involved in the phenylpropanoid biosynthesis as well as identify additional genes that might control this pathway.

## Conflict of interest statement

The authors declare that the research was conducted in the absence of any commercial or financial relationships that could be construed as a potential conflict of interest.

## References

[B1] Agati G. Tattini M. (2010). Multiple functional roles of flavonoids in photoprotection. New Phytol. 186, 786–793 10.1111/j.1469-8137.2010.03269.x 20569414

[B2] Ané J. M. Lévy J. Thoquet P. Kulikova O. de Billy F. Penmetsa V. (2002). Genetic and cytogenetic mapping of DMI1, DMI2, and DMI3 genes of *Medicago truncatula* involved in nod factor transduction, nodulation, and mycorrhization. Mol. Plant Microbe Interact. 15, 1108–1118 10.1094/MPMI.2002.15.11.1108 12423016

[B3] Arrighi J. F. Barre A. Ben Amor B. Bersoult A. Soriano L. C. Mirabella R. (2006). The *Medicago truncatula* lysine motif-receptor-like kinase gene family includes *NFP* and new nodule-expressed genes. Plant Physiol. 142, 265–279 10.1104/pp.106.084657 16844829 PMC1557615

[B4] Broughton W. J. Hernandez G. Blair M. Beebe S. Gepts P. Vanderleyden J. (2003). Beans (*Phaseolus* spp.)—model food legumes. Plant Soil 252, 55–128 10.1023/A:102414671061118055156

[B5] Cooper J. E. (2004). Multiple responses of rhizobia to flavonoids during legume root infection. Adv. Bot. Res. 41, 1–62 10.1016/S0065-2296(04)41001-5

[B6] Dixon R. A. Achnine L. Kota P. Liu C. J. Reddy M. S. S. Wang L. (2002). The phenylpropanoid pathway and plant defence a genomics perspective. Mol. Plant Pathol. 3, 371–390 10.1046/j.1364-3703.2002.00131.x 20569344

[B7] Horváth B. Yeun L. H. Domonkos A. Halász G. Gobbato E. Ayaydin F. (2011). *Medicago truncatula* IPD3 is a member of the common symbiotic signaling pathway required for rhizobial and mycorrhizal symbioses. Mol. Plant Microbe Interact. 24, 1345–1358 10.1094/MPMI-01-11-0015 21692638

[B8] Peer W. A. Murphy A. S. (2007). Flavonoids and auxin transport: modulator or regulators?. Trends Plant Sci. 12, 556–563 10.1016/j.tplants.2007.10.003 18198522

[B9] Poland J. E. Bradbury P. J. Bucler E. S. Nelson R. J. (2011). Genome-wide nested association mapping of quantitative resistance to northern leaf blight in maize. Proc. Natl. Acad. Sci. U.S.A. 108, 6893–6898 10.1073/pnas.1010894108 21482771 PMC3084105

[B10] Reinprecht Y. Yadegari Z. Perry G. E. Siddiqua M. Wright L. C. McClean P. E. (2013). *In silico* comparison of genomic regions containing genes coding for enzymes and transcription factors for the phenylpropanoid pathway in *Phaseolus vulgaris* L. and *Glycine max* L. Merr. Front. Plant Sci. 4:317 10.3389/fpls.2013.00317 24046770 PMC3763686

[B11] Schmutz J. Cannon S. B. Schlueter J. Ma J. Mitros T. Nelson W. (2010). Genome sequence of the palaeopolyploid soybean. Nature 463, 178–183 10.1038/nature08670 20075913

[B12] Schmutz J. McClean P. E. Mamidi S. Wu G. A. Cannon S. B. Grimwood J. (2014). A reference genome for common bean and genome-wide analysis of dual domestication. Nat. Genet. 46, 707–713 10.1038/ng.3008 24908249 PMC7048698

[B13] Tanaka Y. Sassaki N. Akemi O. (2008). Biosynthesis of plant pigments: anthocyanins, betalains and carotenoids. Plant J. 54, 733–749 10.1111/j.1365-313X.2008.03447.x 18476875

[B14] Vogt T. (2010). Phenylpropanoid biosynthesis. Mol. Plant 3, 2–20 10.1093/mp/ssp106 20035037

[B15] Yu O. Jez J. M. (2008). Nature's assembly line: biosynthesis of simple phenylpropanoids and polyketides. Plant J. 54, 750–762 10.1111/j.1365-313X.2008.03436.x 18476876

